# Estimated Cost of Developing a Therapeutic Complex Medical Device in the US

**DOI:** 10.1001/jamanetworkopen.2022.31609

**Published:** 2022-09-14

**Authors:** Aylin Sertkaya, Rebecca DeVries, Amber Jessup, Trinidad Beleche

**Affiliations:** 1 Eastern Research Group Inc, Lexington, Massachusetts; 2 US Department of Health and Human Services, Office of Inspector General, Washington, DC; 3 US Department of Health and Human Services, Office of the Assistant Secretary for Planning and Evaluation, Washington, DC; 4 US Department of Health and Human Services, Office of Science and Data Policy, Washington, DC

## Abstract

**Importance:**

The US medical device market is the world’s largest, but estimates of the cost to bring a medical device to market are not available to help inform policy making and regulatory efforts to enhance device safety and innovation.

**Objective:**

To estimate the mean expected capitalized cost of developing a novel therapeutic complex medical device.

**Design, Setting, and Participants:**

In this economic evaluation, an analytical model of novel therapeutic complex medical device development using data from public and proprietary sources with coverage from 2000 through 2018 was used to estimate the cost, duration, and phase transition success probability associated with each stage of development. Data analysis was completed in September 2021.

**Exposures:**

Conduct of nonclinical and clinical studies; payment of FDA user fees for novel therapeutic complex medical devices.

**Main Outcomes and Measures:**

Mean development cost (in 2018 US dollars) incurred by developers for an FDA-approved novel therapeutic complex medical device, accounting for failures and cost of capital.

**Results:**

In this economic analysis, the mean development cost for a novel therapeutic complex medical device was $54 million (95% CI, $25 million-$200 million) excluding any postapproval studies that might be required. After accounting for the cost of failed studies and cost of capital, the mean capitalized cost of bringing a novel therapeutic complex medical device to the US market was $522 million (95% CI, $205 million-$3382 million). The key factors associated with this cost were the phase transition probabilities: 46.9% for nonclinical to feasibility study, 48.0% for feasibility to pivotal study, 75.7% pivotal study to FDA premarket approval submission, and 80.5% for FDA premarket approval submission to approval. The nonclinical development stage constituted the largest portion of overall cost at 85.0% with the FDA review stage with the highest phase transition probability accounting for only a small fraction at 0.5%.

**Conclusions and Relevance:**

In this economic evaluation study, the cost of therapeutic complex medical device development from proof of concept through postapproval stages was assessed accounting for the cost of failures and the cost of capital. Existing estimates did not account for all stages of development, capitalization, or failure costs, which this study suggests were substantial.

## Key Points

**Question:**

What are the estimated costs associated with bringing a novel therapeutic complex medical device to market in the US?

**Findings:**

In this economic evaluation study using data from public and proprietary sources, an analytical cost model found that the estimated mean expected capitalized development cost per therapeutic complex medical device was $522 million. The nonclinical development stage accounted for 85% of this cost, whereas the US Food and Drug Administration submission, review, and approval stage comprised 0.5%.

**Meaning:**

Results of this economic analysis study provide an estimate of development cost for novel therapeutic complex medical devices using public and proprietary data sources that account for cost of failures and cost of capital.

## Teaser

This economic evaluation study uses an analytical model to estimate the total cost of bringing a novel therapeutic complex medical device to the US market.

## Introduction

There is ongoing debate on how to spur innovation of novel medical products while controlling health care costs. Part of this debate has focused on the rising cost of bringing a medical product to market. The medical product development process is complex and requires collecting safety and effectiveness data to inform regulatory approval for marketing in the US. In 2016, US spending on medical devices and in-vitro diagnostics totaled 5.2% ($173.1 billion) of total national health expenditures, making the US the world’s largest market for medical devices.^1^ While there have been efforts to quantify the total cost of bringing novel drugs to the US market,^2,3^ limited work has been done to estimate the investment needed for a novel medical device.

Given that medical devices range from simple tongue depressors to highly complex implantable closed loop insulin delivery systems, estimating mean development cost is challenging. Thus, we focused on estimating cost associated with developing novel therapeutic complex medical devices (hereinafter, complex devices), a smaller subset. Complex devices are class III devices (eg, implantable cardiac pacemakers, breast implants, and hemodialysis machines) that usually sustain or support life, are implanted, or present potential unreasonable risk of illness or injury, and require the submission of a premarket approval (PMA) application to the US Food and Drug Administration (FDA).^4^ We excluded diagnostic devices that are subject to PMA application requirements for marketing in the US from this analysis and those devices that qualify for the FDA 510(k) clearance route. Given this situation, our subset represents less than 1% of all medical devices approved by FDA per year.^5^

**Figure 1.**
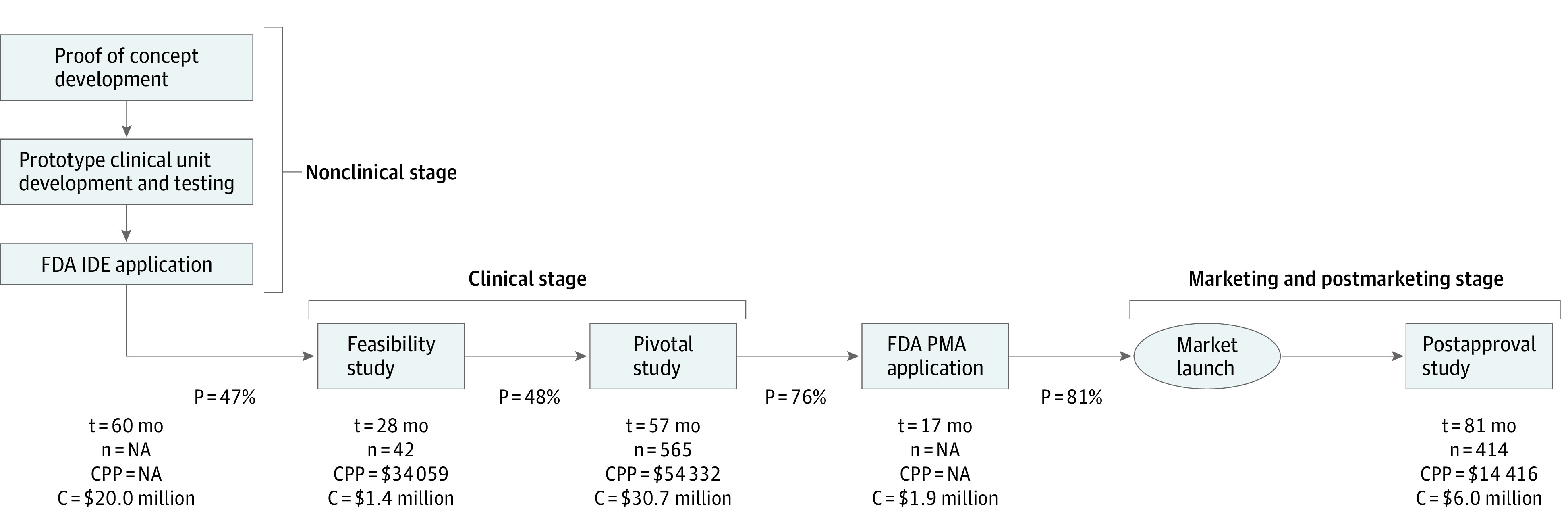
Stages of Therapeutic Complex Medical Device Development In this flow, these are costs that do not incorporate the cost of capital or failure, or removing the phase probabilities. C indicates phase cost (in $ 2018); CPP, cost per patient (in $ 2018); n, number of patients; NA, not applicable; P, phase transition success probability (%); t, phase duration (in months).

The initial stage of complex device development involves the creation of a proof of concept document for a medical need that outlines the steps to determine whether the concept is practical (Figure 1).^6^ The next stage involves development of a device prototype intended for bench and animal testing that may be partially tested on a few patients but not reported in trial registries. In many cases, this stage also includes discussions with FDA under one or more Q-submissions.^7^ On successful completion of the prototype, if the clinical study will be performed in the US (and assuming the clinical study is determined to be a significant risk study), the developer submits an investigational device exemption application to FDA and an institutional review board application to the applicable institutional review board for approval before it can begin clinical studies with human participants. The clinical phase typically includes conducting a feasibility study followed by a pivotal study. Feasibility studies involve a small population of patients to obtain preliminary safety and performance information on the complex device. If results are favorable, the developer undertakes a pivotal study with a larger population of patients to determine the safety and effectiveness of the device. For some PMA applications, multiple feasibility or pivotal studies may be required.

On successful completion of these studies, the developer compiles all the scientific evidence collected from the clinical (and nonclinical) studies to demonstrate reasonable assurance of safety and effectiveness of the device when used in accordance with the indications and submits a PMA application to FDA for review. If the application is approved, the developer can begin marketing its device in the US. The FDA may also request postapproval studies (PASs) to help assure continued safety and effectiveness of the approved device.^5^ Data obtained from PASs supplement performance data included in the PMA application and may uncover important design, mechanical, electrical, and user-related problems not identified in premarket clinical testing.^8^ Over time, these devices may undergo many modifications via the PMA supplement pathway that allows manufacturers to market newer models or expand indications at much lower cost. These postapproval modifications via the PMA supplement are, however, excluded from the scope of this study.

To date, a limited number of published studies evaluate characteristics of different types of medical device studies (eg, pivotal studies, PASs)^9,10^ and estimate costs to developers for various parts of the overall PMA application process.^11,12^ Among the latter group, only 1 study estimated the medical device development cost for small medical device companies^9^ and another estimated the median cost of a PAS.^12^

We developed an analytical model to estimate the total cost of bringing a novel complex device to the US market that accounts for the cost of failures (ie, not successfully completing a given development stage and abandoning development as a result) and cost of capital using public and proprietary data.

## Methods

This economic evaluation study used existing public and proprietary data sources and hence was exempt from institutional review board or ethics committee review per the Common Rule (45 CFR §46). This study followed the relevant portions of the Consolidated Health Economic Evaluation Reporting Standards (CHEERS) reporting guideline for economic evaluations.

### Analytical Model

Using the framework depicted in Figure 1, we constructed an analytical cost model to estimate the mean expected capitalized cost of developing a novel complex device that accounts for the cost, duration, the probability of successfully transitioning from one development stage to the next and the cost of capital (ie, the required rate of return net of inflation for an investor) using the approach by DiMasi et al.^2^ First, we calculated the expected cost of each complex device development stage, which accounts for failures, by dividing the cost estimated for that stage by the aggregate phase-specific probability of the product successfully making it to market. Then, we calculated the mean expected capitalized cost of the same stage by capitalizing the estimated mean expected cost over the duration of that stage as described in DiMasi et al^2^ using a real cost of capital rate of 10.4%^13^ (eTable 2 in the Supplement) for the medical device market. For example, we calculated the expected cost of the feasibility study stage at $4.9 million by dividing the mean cost of conducting a feasibility study estimated at $1.4 million by 29.2%, which is the mean aggregate probability of a complex device successfully making it to market from that development stage. We then calculated the mean expected capitalized cost of the feasibility study stage, which we estimated to last 28.0 months, at $10.0 million, by applying continuous compounding assuming that this cost is distributed uniformly over the 28.0 months. Last, we calculated the mean expected capitalized cost of complex device development by summing the estimated mean expected capitalized cost for each complex device development stage. eMethods in the Supplement include more details.

### Data Sources

We used several data sources to estimate our model parameters including ClinicalTrials.gov, the FDA PMA application and PAS databases, a custom tabulation from proprietary databases, and the professional opinion of 9 federal and industry medical device experts following the snowball method.

**Figure 2.**
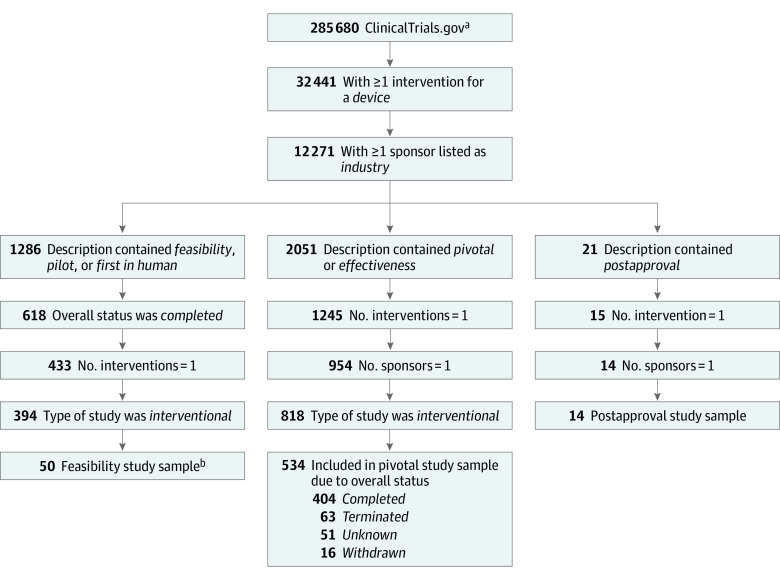
Identification of Feasibility, Pivotal, and Postapproval Studies from ClinicalTrials.gov ^a^Data were extracted from ClinicalTrials.gov on October 30, 2018. ^b^The 50 studies that we could be reasonably sure were, in fact, feasibility studies for medical devices constitute our feasibility study convenience sample. This selection was based on manual review of detailed study descriptions for the 394 *interventional* studies identified in ClinicalTrials.gov.

ClinicalTrials.gov is a registry of privately and publicly funded clinical trials conducted around the world launched in September 2000. The database is updated daily and contains information such as study start/end dates and number of patients enrolled. We downloaded the monthly archived data file on October 30, 2018, which contained 285 680 unique research studies. Of those records, 32 441 studies had at least 1 intervention listed as *device*. We limited our sample to the 12 271 studies that had at least 1 sponsor listed as *industry* to exclude studies that were likely conducted for research purposes only and not intended to support a PMA application. We then used this subset of studies to define our feasibility study (n = 50), pivotal study (n = 534), and PAS (n = 14) samples using various other data fields available in ClinicalTrials.gov (eg, study description, study status, and type of study). We constructed these samples following an approach similar to Rathi et al^9^ (Figure 2).

We used the FDA PMA application and PAS data available on the FDA website.^8,14^ For each approved PMA application, the FDA PMA database lists the submission and approval dates, supporting clinical study data in the Summary of Safety and Effectiveness Data attachment. On September 7, 2018, we downloaded data on a total of 191 original PMAs approved during the period from January 2013 through September 2018, of which 151 were for complex devices. The PAS database provides information on study status and various study protocol parameters (eg, number of patients enrolled, study design, type of data source, such as new data collection, external registry, or sponsor registry), and links each PAS to an approved device by PMA number. We downloaded data on a total of 718 PASs on April 23, 2019. Of these, 322 were for devices approved between 2013 and 2018. Although 139 of these PASs corresponded to 85 (of 151) of the complex device PMAs identified for inclusion in this study, only 109 of these corresponding to 73 PMAs required new data collection by the developer. We therefore limited our PAS data set to these 73 studies. Data analysis was completed in September 2021.

For clinical study cost, we used a custom tabulation from a proprietary database of negotiated investigator grants, outsourcing contracts, and clinical trial sponsors.^15^ These data covered the period 2004 through 2012 and included average expenditures for the full range of cost elements associated with clinical studies (eg, cost of institutional review board approvals, cost of protocols, patient recruitment costs, administrative staff costs). We used available data on the *devices and diagnostics* category to estimate per-patient clinical study costs. Additional information on these data along with assumptions used to extrapolate certain variables are available in Sertkaya et al.^16^

We also interviewed a panel of 9 medical device experts to gain additional insights into the cost, duration, and phase transition success probability associated with the nonclinical phase of complex device development. We aggregated responses to create group averages and conducted follow-up interviews to probe further about nonclinical phase nuances, as needed.

### Model Parameters

Table 1 presents the parameter estimates, assumptions, and data sources for our complex device development cost model. For each study phase, we estimated 6 parameters. The technical supplement contains additional information regarding how (eMethods) and why we used the various sources to estimate each parameter (eMethods, eTable 1, eTable 2 in the Supplement).

**Table 1.** Summary of Therapeutic Complex Medical Device Development Cost Model Parameters and Assumptions

Parameter	Phase	Value	Source
Phase durations (in months)	Nonclinical	60.0	Expert opinion
	Feasibility study	28.0	ClinicalTrials.gov feasibility study and FDA PMA samples
	Pivotal study	56.9	FDA PMA sample
	FDA PMA review	17.4	FDA PMA sample
	Postapproval study	81.2	ClinicalTrials.gov post-approval study sample
Start to start (in months)	Nonclinical to feasibility study	60.0	Expert opinion
	Feasibility study to pivotal study	37.2	ClinicalTrials.gov feasibility study sample
	Pivotal study to FDA PMA submission	42.4	FDA PMA sample
	FDA PMA submission to approval	17.4	FDA PMA sample
No. of patients enrolled	Nonclinical	NA	Not applicable
	Feasibility study	42	ClinicalTrials.gov feasibility study and FDA PMA samples
	Pivotal study	565	FDA PMA sample
	FDA PMA review	NA	Not applicable
	Postapproval study	414	FDA PAS sample
Per-patient cost (in $ 2018)	Nonclinical	NA	Not applicable
	Feasibility study	34 059	Medidata Solutions^15^
	Pivotal study	54 332	Medidata Solutions^15^
	FDA PMA review	NA	Not applicable
	Postapproval study	14 416	Medidata Solutions^15^
Out of pocket costs (in $ 2018)	Nonclinical	20 000 000	Expert opinion
	Feasibility study	1 428 249	Calculation: number of patients enrolled × per-patient cost for feasibility
	Pivotal study	30 672 652	Calculation: number of patients enrolled × per-patient cost for pivotal
	FDA PMA review	1 852 816	AdvaMed^17^
	Postapproval study	5 961 197	Calculation: number of patients enrolled × per-patient cost for postapproval
Transition success probabilities (%)	Nonclinical to feasibility study	46.9	Expert opinion
	Feasibility study to pivotal study	48.0	ClinicalTrials.gov feasibility study sample
	Pivotal study to FDA PMA submission	75.7	ClinicalTrials.gov pivotal study sample (estimated as the ratio of completed studies, 404, to all pivotal studies sampled, 534, assuming all completed studies will proceed to FDA PMA phase)
	FDA PMA submission to approval	80.5	FDA^18^
Aggregate stage-specific transition success probabilities (%)	Nonclinical to approval	13.7	Calculation: nonclinical to feasibility study transition success probability × feasibility study to pivotal study transition success probability × pivotal study to FDA PMA submission transition success probability × FDA PMA submission to approval transition success probability
	Feasibility study to approval	29.2	Calculation: feasibility study to pivotal study transition success probability × pivotal study to FDA PMA submission transition success probability × FDA PMA submission to approval transition success probability
	Pivotal study to approval	60.9	Calculation: pivotal study to FDA PMA submission transition success probability × FDA PMA submission to approval transition success probability
	FDA PMA submission to approval	80.5	FDA^18^ (eTable 1 in the Supplement)
Opportunity cost of capital (%)		10.4	Harrington^13^ (eTable 2 in the Supplement)

Abbreviations: FDA, US Food and Drug Administration; PMA, premarket approval.

In brief, *phase duration* represents the time it takes to complete a given phase of complex device development. *Start to start* represents the elapsed time in months between the start of one development stage (eg, the feasibility study) supporting a PMA application and the start of the next development stage (eg, the pivotal study). The *number of patients enrolled* represents the average number of patients enrolled during a given clinical study (feasibility, pivotal, or PAS). The *per-patient cost* represents the average cost that a sponsor incurs per patient in a clinical study in 2018 dollars. The *transition success probabilities* reflect the probability of a developer successfully moving from one stage of complex device development to the next. For example, if there are 100 complex devices at the feasibility phase and only 30 are successful and subsequently begin pivotal studies, then the transition success probability from feasibility study to pivotal study stage is 30%. Last, the real cost of capital represents the rate of return (net of inflation) that the developer would otherwise be able to earn at the same risk level as the investment in the novel complex device that has been selected. This value varies substantially by developer-specific factors (eg, product portfolio and size of company) and other exogenous factors (eg, economic and regulatory climate for device development projects). According to Harrington,^13^ the estimated value for the medical device sector ranges from 9.2% to 11.4%. We used 10.4% as the real cost of capital (eTable 2 in the Supplement).

### Statistical Analysis

We used nonparametric bootstrapped resampling with replacement method (10 000 iterations) to calculate 95% CIs for the estimates. Statistical analysis was performed using @Risk 8.0.0, Industrial Edition (Palisade Company LLC). Statistical significance was not assessed.

## Results

In this economic evaluation study, we estimated the mean development cost for a novel complex device at $54 million (95% CI, $25 million-$200 million) before conducting PASs, and $60 million (95% CI, $27 million-$209 million) after accounting for PASs (Table 2). The estimated median cost per study was approximately $6.0 million (95% CI, $0-$8 million). For the cost excluding PASs, 37% was nonclinical stage related, 60% was clinical stage (ie, feasibility and pivotal study) related, and the remaining 3% was associated with the FDA PMA stage. When capitalized to account for the cost of capital and after accounting for the cost of failures, expected capitalized mean development cost was estimated at $522 million (95% CI, $205 million-$3382 million) without PASs and $526 million (95% CI, $207 million-$3396 million) with.

**Table 2.** Expected Average Cost of Developing a Therapeutic Complex Medical Device for the US Market^a^

Phase	Probability of FDA approval from given phase (%)^b^	$ (%)			
		Cost (in million 2018 $)^c^	Expected cost (in million 2018 $)^d^	Capitalized cost to date of launch (in million 2018 $)^e^	Expected capitalized costs (in million 2018 $)^f^
Nonclinical	14	20.0 (37)	145.7 (72)	60.8 (57)	442.8 (85)
Clinical (feasibility and pivotal stages)	NA	32.1 (59)	55.2 (27)	43.6 (41)	76.8 (15)
Feasibility study	29	1.4 (3)	4.9 (2)	2.9 (3)	10.0 (2)
Pivotal study	61	30.7 (57)	50.4 (25)	40.6 (38)	66.7 (13)
FDA PMA review	81	1.9 (3)	2.3 (1)	2.0 (2)	2.5 (1)
Postapproval study^g^	NA	6.0 (NA	6.0 (NA	4.3 (NA	4.3 (NA)
Total (without postapproval study costs)	NA	54.0 (100)	203.3 (100)	106.4 (100)	522.1 (100)
Total (with postapproval study costs)	NA	60.0 (NA)	$209.2 (NA)	110.6 (NA)	526.4 (NA)

Abbreviations: FDA, US Food and Drug Administration; NA, not applicable; PMA, premarket approval.

^a^ Values may not add up due to rounding.

^b^ The estimate represents the transition probability from the given phase to approval.

^c^ These estimates represent the cash outlay not adjusted for the cost of capital or failures.

^d^ These estimates represent development cost after adjusting for the cost of failures computed as the total cash outlay divided by the transition success probability. Expected cost includes the cost of failures but not the cost of capital.

^e^ These estimates represent the cost at the point of launch after adjusting for the cost of capital. Capitalized costs include the cost of capital but not the cost of failures.

^f^ Expected capitalized costs include the cost of failures and the cost of capital.

^g^ Postapproval cost includes pivotal study follow-up cost incurred after the PMA is approved. In the current model (without diagnostic devices), however, these follow-up costs are 0.

The key factors associated with this cost were the phase transition probabilities: 46.9% for nonclinical to feasibility study, 48.0% for feasibility to pivotal study, 75.7% pivotal study to FDA premarket approval submission, and 80.5% for FDA premarket approval submission to approval.

From an expected capitalized cost perspective, the share of cost represented by the nonclinical stage is approximately 85%, regardless of whether postapproval cost is included with the FDA review stage with the highest phase transition probability accounting for only a small fraction at 0.5%. This finding means that the nonclinical stage represents the largest portion of total expected capitalized development cost, primarily because the probability of moving from the nonclinical stage to a marketable complex device is only 14%. Yet, as the developer successfully transitions from one stage to another, the likelihood of approval increases. For example, the odds of a complex device making it to market is substantially higher if the device has already cleared the feasibility study stage than one that is at the proof-of-concept development stage.

The clinical stage of complex device development accounts for approximately 60% of total cost, but this share decreases to 15% when the cost is capitalized. The pivotal clinical study stage comprises the largest share of clinical development cost, due primarily to enrolling large number of patients (565 on average vs 42 for feasibility studies), taking twice as long as feasibility studies (57 months vs 28 months), and greater cost (approximately $31 million vs $1.4 million).

## Discussion

To our knowledge, this study represents the only analysis of complex device development cost that captures every development stage for a complex device including postapproval studies and accounts for failures and cost of capital. Other related studies we identified do not consider all stages of development for a medical device or characterize only 1 aspect of development.^11,12^ Further, none of these studies account for costs associated with failures or capital.

Makower et al^11^ surveyed 204 medical device companies—mostly small—and estimated the cost to be $94 million ($119 million in 2018 dollars). Of this cost, approximately 30% was nonclinical phase–related, 50% was clinical phase related and the remaining 20% was for getting FDA PMA approval for marketing the device in the US. The study did not provide cost associated with any PASs, nor did it incorporate the cost of failure or cost of capital, which makes their estimates not directly comparable to ours. The difference in results may be associated with differences in methodology, scope (complex devices vs all devices developed by small medical device companies), and what is included in development costs. For example, Makower et al^11^ included operational costs incurred during FDA review as part of the PMA approval cost whereas our study excluded them. While such costs may be applicable to small device manufacturers with a single product in development, this may not apply for medium to large device manufacturers with an established revenue stream from their currently marketed devices.

Rising and Moscovitch^10^ reported an estimate of study duration (median, 3 years) comparable to ours but a substantially lower estimate for patient enrollment (median, 297 patients) for pivotal studies from their review of 27 approved devices. Rathi et al^9^ also estimated median enrollment for PMA pathway medical device studies and reported different results than ours for 52 feasibility studies (median, 65 patients), 30 pivotal studies (median, 241 patients) and 33 PASs (median, 222 patients).

Wimmer et al^12^ developed a model to estimate the cost of PASs for medical devices using information from integrative structured interviews with 12 domain experts. Applying their model to 277 PASs, they estimated a median cost of approximately $2.2 million ($2.3 million in 2018 dollars) per study compared with our estimate of about $6.0 million.

### Limitations

This study has limitations. First, we were not able to identify and use all clinical studies of complex devices in ClinicalTrials.gov and hence were limited to small convenience samples for which data elements for the listed studies were mostly complete and likely coded correctly. Further, we were unable to identify and eliminate those clinical studies supporting a 510(k) FDA application, which may have resulted in underestimates of such parameters as average number of patients and study duration because 510(k) device studies, when required, may not be as extensive as those required for a novel complex device. Moreover, we could have underestimated phase transition success rates if relevant pivotal studies were launched after the date of data download. Study reporting may have been less consistent during the early years of ClinicalTrials.gov, which could make estimates that include these data less reliable. Nonetheless, our approach to selecting our convenience sample of studies is similar to those used by other researchers who have characterized different features of the various study phases along the PMA pathway.^9^ Second, the nonclinical phase duration and associated cost is based on expert opinion because there are no reliable sources of publicly available information on this early development phase. Expert opinion is subject to multiple heuristic biases including availability, representativeness, and anchoring, among others. While we tried to minimize the potential for such biases by interviewing a small pool of experts, we acknowledge that expert opinion is not a substitute for rigorous empirical methods. Third, it was not possible to further delineate the per-patient clinical study cost for the *devices and diagnostics* category provided by Medidata Solutions. To the extent that clinical trials for some diagnostic devices may be less expensive than those for complex devices, the per-patient cost for feasibility, pivotal, and postapproval studies could be underestimated. Last, the degree of complexity across different complex devices is highly variable. For example, an implantable artificial kidney is likely to be orders of magnitude more costly to develop than a coronary stent. Thus, we expect there to be a large degree of variability around the average development costs estimated here.

## Conclusions

This economic evaluation study is the first analysis, to our knowledge, of complex device development cost through the PAS stage accounting for capital and failure costs. Although complex device development is a costly endeavor, overall, resources expended for development are much lower than those estimated for novel drug development, which is more than 6 times costlier according to other estimates.^2,3^

The complex device development cost estimates presented have the potential to inform policy making and regulatory efforts to enhance device safety and innovation.

## Notes

Supplement. **eMethods.** Calculations and Estimates **eTable 1.** FDA PMA Approvals From 2001 Through August 2018 **eTable 2.** Real Cost of Capital Estimates
