# Supplementary material for: Estimated Cost of Developing a Therapeutic Complex Medical Device in the US
Source: JAMA Netw Open. 2022 Sep 14;5(9):e2231609. doi: 10.1001/jamanetworkopen.2022.31609 (PMC9475382; doi:10.1001/jamanetworkopen.2022.31609)
Supplement: Supplement. — eMethods. Calculations and Estimates eTable 1. FDA PMA Approvals From 2001 Through August 2018 eTable 2. Real Cost of Capital Estimates [file jamanetwopen-e2231609-s001.pdf]

Supplemental Online Content

Sertkaya A, DeVries R, Jessup A, Beleche T. Estimated cost of developing a therapeutic complex medical device in the US. *JAMA Netw Open*. 2022;5(9):e2231609. doi:10.1001/jamanetworkopen.2022.31609

eMethods. Calculations and Estimates

eTable 1. FDA PMA Approvals From 2001 Through August 2018

eTable 2. Real Cost of Capital Estimates

This supplemental material has been provided by the authors to give readers additional information about their work.

© 2022 Sertkaya A et al. *JAMA Network Open*.

eMethods. Calculations and Estimates

The following sections discuss how total expected capitalized cost was calculated and the basis for the model parameter estimates presented in Table 1.

Methods

We calculated the total expected capitalized cost, *E(CC)*, of developing a given complex device following the approach by DiMasi et al as the sum of the expected capitalized cost of each phase *i*:

$$E(CC) = \sum_{i=1}^{n} E(CC_i) \quad (1)$$

where *i* = non-clinical, feasibility study, pivotal study, FDA review, and post-approval study stage and

$$E(CC_i) = \frac{1}{p_i} \int_{t_i^e}^{t_i^b} \left(\frac{C_i}{t_i}\right) e^{rt} dt \quad (2)$$

where *C* is the cash outlay; *r* is the cost of capital that captures the time value effect; *p* is the transition success probability from phase *i* to launch; *t* is the length of the phase; t_i^b is the time from the beginning, *b*, of the given phase to product launch, and t_i^e is the time from the end, *e*, of the given phase to product launch. Equation 2 then becomes:

$$CC_i = \frac{(C_i/t_i)}{r} \left(e^{rt_i^b} - e^{rt_i^e}\right) \quad (3)$$

Parameters

Phase Durations

The phase duration parameter refers to the time it takes to complete a given stage of development depicted in Figure 1. For the non-clinical stage, our estimate of 60 months represents the time it takes for proof of concept development, clinical unit development, and obtaining an IDE, which is required by FDA to test the safety and effectiveness of unapproved medical devices in human subjects.

We derived our feasibility study phase estimate of 28 months by combining the 50 feasibility studies from our ClinicalTrials.gov sample with the 29 feasibility studies from our FDA PMA database sample, removing the duplicate studies (one study), and taking the average feasibility study duration from the combined sample.

Our pivotal and FDA PMA review duration estimates were derived using study duration data from the FDA PMA database. Our post-approval stage duration estimate of 81.2 months is based on the average study duration from our sample of 14 post-approval studies we identified in ClinicalTrials.gov. We had to rely on the ClinicalTrials.gov PAS sample because the FDA PAS database did not publicly report the start and end dates for the completed PASs they have listed.

Time from Phase Start to Next Phase Start

The start to start parameter refers to the elapsed time between the start of one development stage (e.g., feasibility study phase) supporting a PMA application and the start of the next development stage (e.g., pivotal study phase) supporting the same application. For the non-clinical stage to feasibility study phase estimate, we used the same 60-month interval estimated for the non-clinical phase duration; that is, we assumed feasibility testing will begin immediately upon successful completion of the non-clinical development phase.

For the feasibility study stage to pivotal study stage estimate of 37.2 months, we used the ClinicalTrials.gov feasibility study sample. First, we matched the feasibility studies in our sample with pivotal studies in our database based on an examination of the device names, descriptions, and sponsors. Because not all feasibility studies successfully proceed to the pivotal phase, we were able find matching pivotal studies for 48 percent (24 out of 50) of studies in our feasibility sample. Next, using the study start date field, we computed the difference between the start date for the feasibility study and the start date of the pivotal study in our matched sample (24 studies). The average time from feasibility phase start to pivotal phase start represents the average value for these 24 studies in our matched sample.

To calculate the time from pivotal-phase start to FDA PMA submission we relied on our FDA PMA sample of 151 unique PMAs of which 149 had pivotal study data reported in their Summary of Safety and Effectiveness Data (SSED) attachments corresponding to a

total of 209 pivotal studies.[1] We computed the difference in the reported FDA PMA submission date and pivotal study start date for each of the 209 pivotal studies. The average time from pivotal study start date to FDA PMA submission date, 42.4 months, represents the average value for these 209 studies in our FDA PMA sample.

Similarly, we relied on our FDA PMA sample that consists of 151 PMA approvals with information on PMA submission and approval dates to estimate the average time it takes from FDA PMA submission to PMA approval (17.4 months).

Average Number of Patients Enrolled

The patient enrollment parameter represents the average number of patients enrolled for a given clinical study (feasibility or pivotal) stage supporting a PMA application which is one of the key drivers of the cost of a clinical study. We derived our feasibility study estimate of 27.47 patients using the 78 studies from our combined feasibility and FDA PMA samples. Next, we estimated that on average, a developer conducts 1.53 feasibility studies per PMA based on the 19 PMAs that provided information on the feasibility studies conducted in their SSEDs. This translated to an estimate of 42 patients (≅ 27.47 × 1.53) for the feasibility study stage.

For our pivotal study stage estimate, we first averaged the number of patients enrolled in the 209 pivotal studies as reported in our FDA PMA sample to estimate the number of patients per pivotal study at 402.47. Next, we estimated that on average, a developer conducts 1.4 pivotal studies per PMA based on the 149 PMAs that provided information on the pivotal studies conducted in their SSEDs. This translated to an estimate of 565 patients (≅ 402.47 × 1.40) for the pivotal study phase.

We used the FDA PAS data to estimate the average number of patients for the PAS stage. Of the 151 PMAs in our sample, FDA requested post-approval studies that require collection of new data by the developer at the time of PMA approval for 73 PMAs.[2] However, only 67 of the 73 PMAs contained planned patient enrollment data for the associated PASs. The average number of planned patient enrollment for the PAS phase among the 67 PMAs with associated PASs was 895 patients. Given the 46 percent probability of FDA requiring a PAS involving new data collection from a sponsor at the time of PMA approval,[3] we estimated the average number of patients for the PAS phase at 414 (≅ 895 × 0.46).

---

[1] Some of the 149 PMA submissions included information on more than one pivotal study. Of the 149 PMAs with supporting pivotal study information, 79% reported one pivotal study, 13% reported two, and the remaining 8% reported three or more pivotal studies.

[2] Device developers incur costs for those PASs that do not require new data collections, which are expected to cost lower than those that have this requirement.

[3] Computed as the ratio of 67 PMAs with PAS requirement involving new data collection and reported number of planned patients to 145 PMAs (= 151 PMAs – 6 PMAs without reported number of planned patients for their PASs).

Average Per-Patient Cost

The per-patient cost parameter represents the average cost a sponsor incurs per-patient in a clinical study supporting a PMA application. Our per-patient cost estimates are based on the estimates we had obtained from Medidata Solutions, for the “devices and diagnostics” therapeutic area.

We used the Phase 1 per-patient cost reported by Medidata Solutions as an estimate for complex device feasibility study per-patient cost. We assumed that the per-patient cost for a complex device pivotal study is equivalent to the average of Phase 2 and Phase 3 per-patient cost reported by Medidata Solutions. For our PAS per-patient cost estimate, we used $14,416 reported by Medidata Solutions for a Phase 4 study.

Cost by Stage of Development

The cost parameter represents the average expenses (not adjusted for failures or cost of capital) a developer incurs during a given complex device development stage. Our literature review did not identify any studies that report expenditures by stage for complex devices. We relied on expert opinion provided by the interviews we conducted with federal and industry medical device experts to estimate the cost for non-clinical stage at $20 million. The experts interviewed reported a range of costs for this stage with $15 million for the lower bound for less complex therapeutic devices with a clear development path to as high as $35 million for those that are highly complex innovative implantable devices.

We estimated the cost for the feasibility, pivotal, and PAS stages as the product of average number of patients enrolled (42 for a feasibility, 565 for a pivotal, and 895 for a PAS when such a study is required[4]) and per-patient cost ($34,059 for a feasibility, $54,332 for a pivotal, and $14,416 for a PAS) at $1.4 million, $30.7 million, and $6.0 million,[5] respectively.

Our FDA PMA review phase cost estimate of approximately $1.9 million is based on AdvaMed’s 2014 docket submission for FDA’s proposed rule on Medical Device Classification Procedures (FDA-2013-N-1529).[15] In its submission, AdvaMed noted that

[4] The model uses the expected number of patients enrolled for a PAS which is the product of 895 patients per study and the 46 percent chance that a PAS would be required by FDA; 414 (≅ 895 × 0.46).
[5] According to Wimmer et al. [10] the median cost of a PAS study is $2.22 million ($2.15 million in $ 2016) with a range of $1.38 million to $12.78 million based on estimates generated by a panel of 12 medical device experts. Since FDA on average requires 1.5 PAS studies per PMA for those PMAs deemed to need a PAS, this translates to a median cost of $3.33 million for the PAS stage, roughly half of what we estimated using FDA data on PAS study enrollment and per-patient cost information from Medidata Solutions databases.

“total average costs of supporting a PMA” would include $900,000 for panel meeting preparation, $475,000 for PMA submission preparation, $125,000 for pre-approval inspection, and additional costs for MDUFA user fees estimated as the average of $322,147 standard MDUFA fee and $80,537 small business MDUFA fee.[17] Inflating these 2014 figures to 2018 dollars, we estimated the cost for the PMA review phase at around $1.9 million.

Phase Transition Success Probabilities

The phase transition success probability parameter represents the probability of a developer successfully moving from one stage of complex device development to the next. For example, if there are 100 complex devices at the feasibility stage and only 30 of these complex devices successfully complete their feasibility studies and subsequently begin pivotal studies, then our transition success probability from feasibility study stage to pivotal study stage is 30% (= 30 ÷ 100).

Given the lack of publicly available information on the non-clinical stage of development, we relied on expert opinion elicited from interviews with medical device experts to estimate the probability of successfully transitioning from the non-clinical stage to the feasibility stage at 47%, which represents the simple average of estimates ranging from 15 to as high as 90% provided by the nine medical device experts we interviewed. The estimate is intended to represent the transition probability for a single investigational device design and does not capture the iterative nature of the early development stage for complex devices where the developer might revise the design of the prototype, intended use, or other characteristics.

We estimated the transition probability from the feasibility study stage to pivotal study stage as the ratio of our feasibility sample subset with matching pivotal studies (24 studies total) to the full feasibility sample of 50 studies to be 48% (= 24/50).

To estimate the probability of successfully transitioning from the pivotal-study stage to FDA PMA submission stage, we again relied on ClinicalTrials.gov data, but employed a different methodology. We identified 534 pivotal medical device studies in ClinicalTrials.gov. Of these 534 studies, 63 were terminated, 16 were withdrawn, 51 had unknown resolutions, and 404 were completed. First, we assumed that the completed studies are successful in demonstrating the safety and effectiveness of the medical device being investigated. We further assumed that 100% of such successful studies is used in support of a PMA application. Thus, we estimated the probability of successfully transitioning from the pivotal study stage to the FDA PMA submission stage as the ratio of the 404 completed studies to the total sample of 534 at 75.7%.

Our FDA PMA submission to PMA approval transition success probability of 80.5% (i.e., the percent of original PMA applications submitted to FDA that is approved) is based on MDUFA IV CDRH performance data as of August 2018 reported by FDA.[16] MDUFA quarterly updates report the percent of original PMAs approved by CDRH from

2001 to August 2018 annually (Table 1). We used the historical average of 80.5% (with a median of 82%) to represent the FDA submission to approval probability.

Cost of Capital

The real (i.e., net of inflation) cost of capital represents the rate of return that the developer would otherwise be able to earn at the same risk level as the investment in the novel complex device that has been selected. This value varies significantly by sponsor-specific factors, such as product portfolio and size of company, as well as other exogenous factors, such as economic and regulatory climate for device development projects. According to a study by Harrington,[13] the estimated real cost of capital for the medical device sector ranges from a low of 9.2% to a high of 11.4%. We used 10.4% as the average real cost of capital in our model.

Commented [ASPE-TB1]: Revised to be consistent with terminology used in the manuscript.

Commented [ASPE-TB2]: Revised to be consistent with the most current version of the proof. May want to cross-link it with the eRefernce below, i.e., Harrington (2012)[1]

Table 1. FDA PMA Approvals from 2001 through August 2018 (Downloaded from FDA Website on November 9, 2018)

**Year**	**Percent of Original PMAs Approved [a]**
2001	82.0%
2002	72.0%
2003	86.0%
2004	82.0%
2005	90.0%
2006	82.0%
2007	76.0%
2008	81.0%
2009	68.0%
2010	59.0%
2011	71.0%
2012	70.0%
2013	85.0%
2014	86.0%
2015	95.0%
2016	89.0%
2017	91.0%
2018	84.0%
**Mean**	**80.5%**
**Median**	**82.0%**
**Standard Deviation**	**9.1%**

[a] From Quarterly Update on Medical Device Performance Goals - MDUFA IV CDRH Performance Data - Action through 30 June 2018
[b] Reflects 8 months of data in 2018.

Table 2. Real Cost of Capital Estimates from Harrington (2012)[1]

Estimation Method	Firm Size	Study Period	Sample Size	Real Cost of Capital
CAPM [a]	All	2001-2005	44	9.6%
	All	2006-2008	42	11.2%
	Large	2001-2005	12	9.2%
	Large	2006-2008	15	10.9%
	Small	2001-2005	32	9.8%
	Small	2006-2008	27	11.4%
**Average**				**10.4%**

[a] CAPM = Capital asset pricing model

eReference

Harrington SE. Cost of Capital for Pharmaceutical, Biotechnology, and Medical Device Firms. In: Nicholson S, Danzon PM, eds. The Oxford Handbook of the Economics of the Biopharmaceutical Industry. Oxford University Press; 2012.
